# Leukotoxin and pyrogenic toxin Superantigen gene backgrounds in bloodstream and wound *Staphylococcus aureus* isolates from eastern region of China

**DOI:** 10.1186/s12879-018-3297-0

**Published:** 2018-08-13

**Authors:** Chunyan He, Su Xu, Huanqiang Zhao, Fupin Hu, Xiaogang Xu, Shu Jin, Han Yang, Fang Gong, Qingzhong Liu

**Affiliations:** 1 Department of Clinical Laboratory, Shanghai General Hospital, Shanghai Jiaotong University School of Medicine, Shanghai, 200080 China; 2 Institute of Antibiotics, Huashan Hospital, Fudan University, Shanghai, 200080 China; 3 Experimental Research Center, Shanghai People’s Hospital of Putuo District, Shanghai, 200080 China; 4 Department of Clinical Laboratory, the Third Hospital Affiliated to Nantong University, Wuxi, 226000 China

**Keywords:** *Staphylococcus aureus*, Leukocidin gene, Pyrogenic toxin superantigen gene, *lukED* expression, Genetic characteristic

## Abstract

**Background:**

The bicomponent leukotoxins and the pyrogenic toxin superantigens (PTSAgs) are important virulence factors of *Staphylococcus aureus*. It is necessary to survey the prevalence and expression of these toxin-encoding genes for understanding the possible pathogenic capacity of *S. aureus* to cause disease.

**Methods:**

Five leukotoxin genes and thirteen PTSAg determinants were detected for 177 *S. aureus* isolates from blood (*n* = 88) and wound (*n* = 89) infections by Polymerase Chain Reaction (PCR). The expression of leukotoxin ED (*lukED*) was determined by quantitative real-time PCR (qRT-PCR). The genetic backgrounds of isolates were analyzed by Staphylococcal Cassette Chromosome *mec* (SCC*mec*) typing (for methicillin-resistant *S. aureus* isolates), Pulsed-Field Gel Electrophoresis (PFGE), accessory gene regulator (*agr*) typing and Multilocus Sequence Typing (MLST, for representative isolates based on PFGE type) methods.

**Results:**

99.4% (176/177) isolates contained at least one of leukotoxin genes. Among them, 94.9% (168/177), 81.4% (144/177) and 67.8% (120/177) isolates harbored *hlgBC*, *lukED* and *lukAB*, respectively. Compared to leukotoxin genes, there was a relatively lower overall prevalence of PTSAg genes [99.4% versus 72.9% (129/177), *P* < 0.001], and they were organized in 59 patterns, with the most common combination of the *egc* cluster with or without other PTSAg genes. Genetic analysis showed the distributions of certain toxin genes were associated with the genetic backgrounds of isolates. The *egc* cluster was a common feature of CC5 isolates, among which ST5 and ST764 isolates harbored more PTSAg genes. The *lukED* was not present in ST398 isolates, and its expression was quite different among isolates. No significant correlations were observed between the *lukED* expression levels of strains and the ST or *agr* types.

**Conclusions:**

The present study elucidated the distribution of leukotoxin and PTSAg genes and the expression of *lukED* in blood and wound isolates, and analyzed the relationship between them with genetic characteristics of isolates. These data improve the current understanding of the possible pathogenicity of *S. aureus*.

**Electronic supplementary material:**

The online version of this article (10.1186/s12879-018-3297-0) contains supplementary material, which is available to authorized users.

## Background

*Staphylococcus aureus* (*S. aureus*) is a serious pathogen that causes various clinical infections with considerable morbidity and mortality due to its capability to produce different virulence factors [1]. Among these virulence factors, the bicomponent leukotoxins and the pyrogenic toxin superantigens (PTSAgs) have attracted great attention for their ability to destruct the membranes of host cells or regulate the immune responses by activating immune cells abnormally [2–5].

In *S. aureus*, seven leukotoxins have been identified. Panton-Valentine leukocidin (PVL), gamma (γ)-hemolysin (HlgAB and HlgBC), leukotoxin ED (LukED), and leukotoxin AB/GH (LukAB/GH) are found in isolates associated with human infections [2, 3]. Leucocidin MFʹ (LukMFʹ) and leucocidin PQ (LukPQ) are only detected in strains from zoonotic infections [6, 7]. Except for *pvl* (encoding PVL), the data on the overall prevalence of leukotoxin family in clinical *S. aureus* isolates are very limited in China, especially that of the recently identified LukAB, which is the only leukotoxin known to enhance the survival of *S. aureus* [2, 3, 8].

Previous epidemiological data showed that *lukED* existence is widespread among *S. aureus* isolates [9], and this toxin has an important role in *S. aureus* bloodstream infection, impetigo and antibiotic-associated diarrhea [3, 10]. Said-Salim et al. [11], Boakes et al. [12] and Yu et al. [13] reported that the production of PVL differentiated from strain to strain, and this difference is associated with the severity of specific infections (such as skin and soft tissue infections, SSTIs). Then, is the expression pattern of *lukED* among clinical *S. aureus* isolates the same as that of PVL? Until now, no data can be used to clarify this question.

*S. aureus* can also secrete an array of pyrogenic toxin superantigens (PTSAgs), including toxic shock syndrome toxin-1 (TSST-1), staphylococcal enterotoxins (SEs), and SE-like toxins. PTSAgs are able to activate T-cells and antigen-presenting cells (APCs) to release proinflammatory cytokines, increase sensitivity to bacterial lipopolysaccharide (LPS) [14], and are associated with some diseases, such as toxic shock syndrome, food poisoning and allergic syndromes [15]. Study also indicates that PTSAgs can play a major role in the pathophysiological mechanism of sepsis [16]. Therefore, it is required to get better understanding of the PTSAg genes distribution in *S. aureus* isolates from clinical samples.

In this study, we conducted a retrospective study to determine the distribution of genes encoding leukotoxins and PTSAgs among clinical *S. aureus* isolates obtained from blood and wounds. Subsequently, we analyzed the genetic characteristics of these isolates, and the expression of *lukED* in some *lukED*-positive strains. Furthermore, the relationship between genetic backgrounds and the carriage of virulence genes, or the expression level of *lukED* was assessed.

## Methods

### Study design and strain identification

**Fig. 1:**
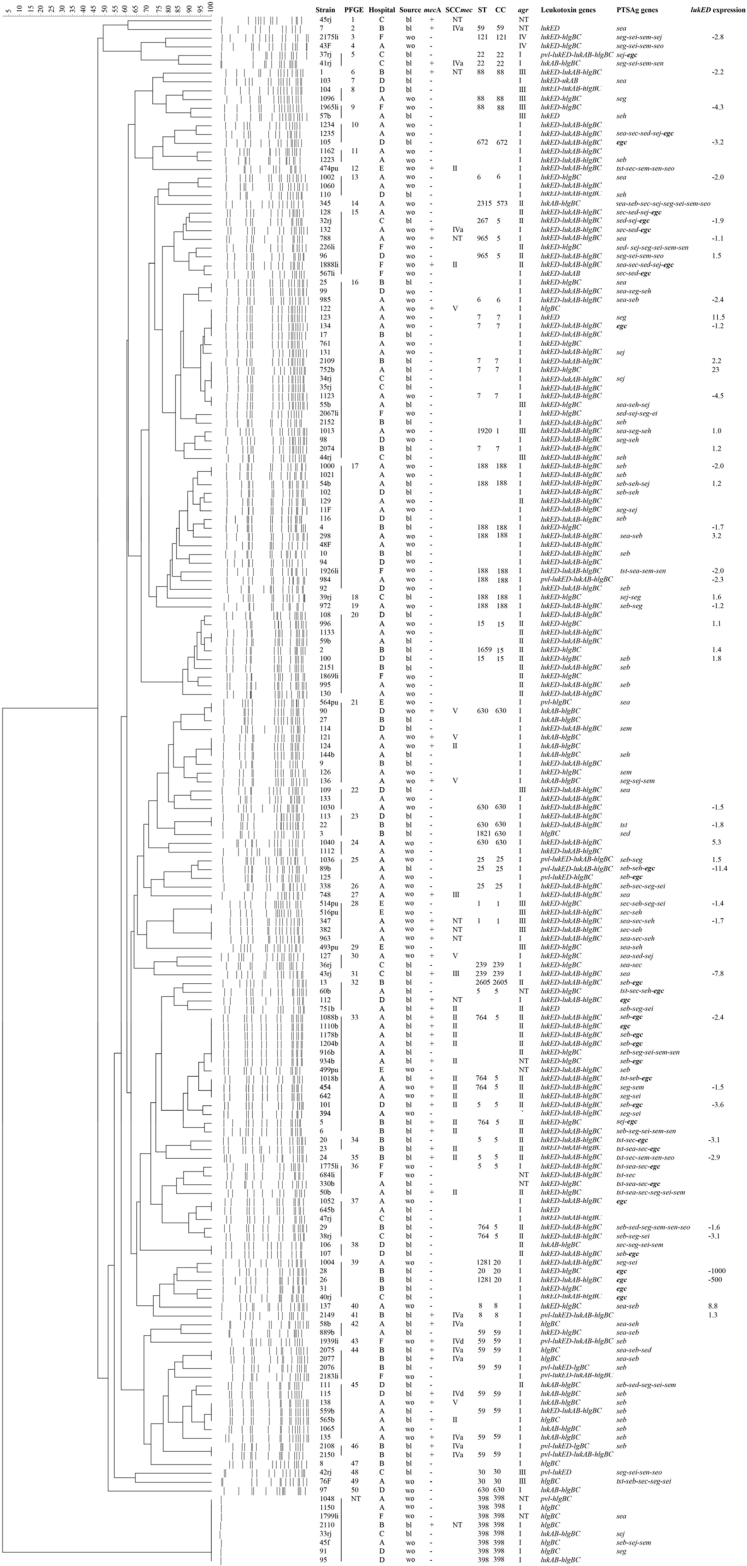
PFGE-based dendrogram showing the genetic relationships of 177 *S. aureus* isolates. PFGE cluster was assigned by ≥ 80% genetic similarity. The hospital origin, source, methicillin resistance, SCC*mec* type, ST type, clonal complex, *agr* type, toxin gene content and the expression of *lukED* of the isolates were showed. NT, not typeable; wo, wound; bl, blood; *egc*, *seg-sei-sem-sen-seo*. *mecA* +, methicillin-resistant *Staphylococcus aureus*; *mecA*-, methicillin-susceptible *Staphylococcus aureus*; PTSAg, pyrogenic toxin superantigen; *lukED* expression, the fold change of *lukED* expression level compared to that of Newman strain, A, Shanghai General Hospital; B, Ruijin Hospital; C, Renji Hospital; D, Shanghai Sixth People’s Hospital; E, Shanghai People’s Hospital of Putuo District; F, The General Hospital of Lishui City, Zhejiang Province

Between June 2014 and October 2016, a total of 177 non-duplicate clinical *S. aureus* isolates (88 strains from blood and 89 isolates from wounds) were separated from six hospitals in, China, namely, Shanghai General Hospital (23 blood isolates and 61 wound isolates, from June 2015 to June 2016), Ruijin Hospital (33 blood isolates, from July 2015 to December 2015), Renji Hospital (15 blood isolates, from January 2016 to March 2016), Shanghai Sixth People′s Hospital (17 blood isolates and 9 wound isolates, from June 2015 to March 2016), Shanghai People′s Hospital of Putuo District (6 wound isolates, from January 2015 to February 2015), and The General Hospital of Lishui City, Zhejiang Province (13 wound isolates, June 2014 to August 2014) (Fig. 1). All of the isolates were identified by VITEK microbiology analyser (bioMérieux, Marcy l′ Etoile, France). Methicillin resistance was confirmed by disk diffusion test with a 30 μg cefoxitin disk (Oxoid, Basingstoke, UK) [17] and Polymerase Chain Reaction (PCR) amplification of *mecA* and *mecC* genes (the primers were listed in Additional file 1) [18, 19]. *S. aureus* Newman was used as a control standard for the amplification and expression of *lukED*. The quality control strain of disk test was ATCC25923. Methicillin-resistant *S. aureus* (MRSA) strains NCTC10442, N315, 85/2082, JCSC4744 and D12 [20] were utilized as reference strains for SCC*mec* type I, II, III, IV and V, respectively.

### DNA extraction

Suspensions of *S. aureus* cultures were incubated with lysostaphin (Sangon, Shanghai, China) at 37 °C for 30 min. Then the genomic DNA was extracted from each isolate using TIANamp Bacteria DNA Kit (Tiangen, Beijing, China) following the manufacturer’s instructions, and used as an amplification template for PCR.

### Detection of leukotoxin and PTSAg genes

All isolates were screened for the presence of genes encoding leukotoxins (*pvl*, *lukAB*, *lukED*, *hlgCB, lukM*) and PTSAgs (*sea-see, seg-sej, sem-seo* and *tst*) by PCR [21–24]. Primers used for the amplification of the toxin genes were listed in Additional file 1. One randomly picked PCR product for each gene was sequenced to verify the certainty of target fragment.

### Genotyping methods

#### SCC*mec* typing

Staphylococcal Cassette Chromosome *mec* (SCC*mec*) type I-V were determined by multiplex PCR using the primers (see Additional file 1) derived from the published sequences for MRSA isolates [25].

#### *agr* typing

PCR amplification of the accessory gene regulatory (*agr*) alleles (allele I to IV) was carried out by a previously described method [26] using the primers shown in Additional file 1.

#### PFGE typing

SmaI-Pulsed-Field Gel Electrophoresis (PFGE) analysis was performed for all isolates to understand their homology as described by McDougal et al. [27]. The patterns of DNA fingerprint were analyzed using BioNumerics sofeware7.0. Percent similarities were identified as described previously [27]. The cluster cutoff was set at 80% similarity.

#### MLST typing

Multilocus Sequence Typing (MLST) was performed by PCR amplification and sequencing of internal fragments of seven housekeeping genes (*arcC*, *aroE*, *glpF*, *gmk*, *pta*, *tpi* and *yqiL*) on representative isolates of each PFGE type as previously outlined by Enright et al. [28]. Sequence types (STs) were determined through the MLST website for *S. aureus* (http://saureus.mlst.net/). The eBURST v.3 [29] was used to classify the related STs into clonal complexes (CCs).

### Expression level of *lukED* at different growth phases

Two clinical *S. aureus* isolates chosen randomly and well-characterized strain Newman were selected to explore the expression variation of *lukED* at early, middle and late exponential growth phases at 37 °C in Tryptic Soy Broth (TSB) (Oxoid, Basingstoke, UK). Total RNA was extracted by TaKaRa MiniBEST Universal RNA Extraction Kit (Takara, Dalian, China) and then used for the synthesis of cDNA with reverse transcription Enzyme Mix (Takara, Dalian, China). The expression of *lukED* was detected by quantitative real-time PCR (qRT-PCR) using SYBR Premix Ex Taq (Takara, Dalian, China) on a 7500 Real-Time PCR system (ABI Biosystems, Cary, USA), as described by previous study [30]. The gene 16s rRNAwas selected as an endogenous control. Specific primers were listed in Additional file 1. The mRNA product of *lukED* in each strain was standardized to strain Newman. 2^-∆∆CT^ method was applied to analyze the relative expression of each strain.

### Transcription level of *lukED* among isolates with different genetic characteristics

The randomly selected *lukED*-positive *S. aureus* isolates [identified further by matrix-assisted laser desorption/ ionization-time of flight mass spectrometry (MALDI-TOF MS, Microflex LT, Bruker Daltonik, Bremen, Germany)] with various PFGE-*agr*-ST types were normally grown to the post-exponential phase of growth at 37 °C in TSB. The expression of *lukED* was performed by qRT-PCR as described above.

### Statistical analysis

Categorical variables were tested for differences between groups using the Pearson’s Chi-square test or Fisher’s exact test. Kruskal-Wallis test was applied to compare the *lukED* expression level of *S. aureus* with different genetic backgrounds. Statistical analysis was computed using IBM SPSS Statistics, Version 23.0 (IBM Corp., Armonk, USA). *P* values less than 0.05 (two-tailed) were considered to be statistically significant.

## Results

### MRSA identification and SCC*mec* typing

Among 177 *S. aureus* isolates, 47 (26.6%) were identified as MRSA (Fig. 1). The prevalence of MRSA isolates was similar in both blood (31.8%, 28/88) and wound isolates (21.3%, 19/89) (*P* = 0.149). Four SCC*mec* types (type II to V) were identified among 39 (83.0%, 39/47) MRSA isolates. An additional 8 (17.0%, 8/47) isolates could not be typed. SCC*mec* II was the predominant type (40.4%, 19/47), followed by SCC*mec* IV (25.6%, 12/47) with subtypes IVa (10 isolates) and IVd (2 isolates), SCC*mec* V (12.8%, 6/47) and SCC*mec* III (4.3%, 2/47). Within MRSA isolates from blood, SCC*mec* II was the most prevalent (50%, 14/28), followed by SCC*mec* IV (32%, 9/28), whereas SCC*mec* V (32%, 6/19) was the most common in wound MRSA isolates.

### *agr* typing

Four *agr* types were identified in 168 isolates, and an additional 9 isolates (5 from blood and 4 from wounds) were untypable (Fig. 1). The *agr* I was the predominant type (63.3%, 112/177), followed by *agr* II (21.5%, 38/177) and *agr* III (9.0%, 16/177). Only two isolates from wounds were found to carry *agr* IV.

### PFGE

The PFGE analysis discriminated 169 *S. aureus* isolates into 50 clonal types, indicating the relatedness of *S. aureus* isolates from six hospitals was relatively diverse (Fig. 1). Six isolates from wounds and two isolates from blood (consisted of one MRSA and seven MSSA) could not be typed using SmaI restriction enzyme. Overall, the most prevalent clonal type, type 16 (11.9%, 21/177), was identified in 10 blood isolates and 11 wound isolates. The second most frequently type was type 17 (8.4%, 15/177), and was found in 5 strains from blood and 10 strains from wounds, followed by type 33 (7.9%, 14/177), and type 20 and 21 (5.7%, 10/177 each). The remaining 45 types were showed in Fig. 1.

### MLST

MLST was performed on 72 representative isolates of each PFGE pattern (randomly selected) and 8 non-PFGE typeable isolates. As shown in Fig. 1, a total of 27 distinct STs were identified. The most common ST type was ST59 (11.3%, 9/80), with 7 strains from blood and 2 from wounds, followed by ST188 and ST398 (10.0%, 8/80 each). Interestingly, 8 non-PFGE typeable isolates were all identified as ST398, including 2 blood isolates and 6 wound isolates (1 MRSA and 7 MSSA). The STs comprised of six isolates (7.5%, 6/80 each) were ST7 and ST764. ST5 and ST630 were found in five isolates (6.3%, 5/80), respectively. In MRSA and MSSA isolates, the most frequently detected ST types were ST59 and ST188, respectively. Moreover, ST59 and ST398 were the predominant types in blood and wound isolates, respectively. By eBURST analysis, CC5 was the most common clone (17.5%, 14/80), followed by CC59 (11.3%, 9/80), CC188 and CC398 (10%, 8/80 each) (Fig. 1).

### Distribution of leukotoxin determinants

The data on the prevalence of leukotoxin genes were summarized in Table 1 and Fig. 1. Overall, *hlgBC* was the most prevalent (94.9%, 168/177), followed by *lukED* (81.4%, 144/177), *lukAB* (67.8%, 120/177) and *pvl* (7.9%, 14/177). All isolates were *lukM* negative. No significant difference was observed with respect to the overall possession of leukotoxin genes between blood and wound isolates.

**Table 1:** Distribution of leukotoxin and PTSAg genes among *S. aureus* isolates from blood and wounds

Gene	No. of isolates positive for the gene [% of total (*n* = 177)]	No. of isolates positive for the gene in two origins (%)		*P* value	No. of isolates positive for the gene in MRSA and MSSA (%)		*P* value
		Blood (*n* = 88)	Wounds (*n* = 89)		MRSA (*n* = 47)	MSSA (*n* = 130)	
Leukotoxin gene							
*lukED*	144 (81.4)	73 (83.0)	71 (80.0)	0.587	32 (68.1)	112 (86.2)	**0.006**
*lukM*	0	0	0	NA	0	0	NA
*hlgCB*	168 (94.9)	81 (92.0)	87 (97.8)	0.099	44 (93.6)	124 (95.4)	0.701
*lukAB*	120 (67.8)	56 (63.6)	64 (71.9)	0.239	33 (70.2)	87 (66.9)	0.679
*lukPV* (*pvl*)	14 (7.9)	7 (7.9)	7 (8.0)	0.982	4 (8.5)	10 (7.7)	1.000
PTSAg gene							
*tst*	13 (7.3)	8 (9.1)	5(5.6)	0.376	5 (10.6)	8 (6.2)	0.335
*sea*	33 (18.6)	14 (15.9)	19 (21.3)	0.353	13 (27.7)	20 (15.4)	0.220
*seb*	50 (28.2)	30 (34.1)	20 (22.5)	0.086	16 (34.0)	34 (26.2)	0.303
*sec*	25 (14.1)	9 (10.2)	16 (18.0)	0.139	10 (21.3)	15 (11.5)	0.100
*sed*	13 (7.3)	5 (5.7)	8 (9.0)	0.399	4 (8.5)	9(6.9)	0.748
*see*	0	0	0	NA	0	0	NA
*seg*	65 (36.7)	33 (37.5)	32 (36.0)	0.831	18 (38.3)	47 (36.2)	0.794
*seh*	19 (10.7)	10 (11.4)	9 (10.1)	0.788	4 (8.5)	15 (11.5)	0.566
*sei*	52 (29.4)	31 (35.2)	21 (23.6)	0.089	16 (34.0)	36 (27.7)	0.413
*sej*	20 (11.3)	8 (9.1)	12 (13.5)	0.356	4 (8.5)	16 (12.3)	0.481
*sem*	51 (28.8)	31 (35.2)	20 (22.5)	0.061	18 (38.3)	33 (25.4)	0.094
*sen*	40 (22.6)	28 (31.8)	12 (13.5)	**0.001**	15 (31.9)	25 (19.2)	0.075
*seo*	38 (21.5)	25 (28.4)	13 (14.6)	**0.025**	13 (27.7)	25 (19.2)	0.576

*P* < 0.05 was considered statistically significant are captured in bold

*NA* not available

With the exception of one MRSA (PFGE1-MRSA-SCC*mec* NT-*agr* NT) without toxin gene detected from blood, a total of 176 isolates (99.4%) harbored 446 leukotoxin genes forming 10 distinct leukotoxin gene profiles (Fig. 1). The most common combination was *lukED*, *lukAB* plus *hlgBC* (52.5%, 93/177), followed by *lukED* plus *hlgBC* (18.1%, 32/177) and *lukAB* plus *hlgBC* (9.6%, 17/177). Among all isolates detected, eight (4 blood and 4 wound isolates, 4.5%, 8/177) harbored the most leukotoxin genes content (*lukED*, *lukAB*, *hlgBC* and *pvl*), including 3 MRSA (SCC*mec* IV) and 5 MSSA. Moreover, 13 isolates (7.3%, 13/177) were only *hlgBC* positive and 5 isolates (2.8%, 5/177) harbored only *lukED*. The average possession of leukotoxin genes between MRSA and MSSA (2.40 versus 2.56 per strain) was similar, whereas, an analysis on single leukotoxin gene revealed that *lukED* was significantly more common in MSSA than in MRSA (86.2% versus 68.1%, *P* = 0.006).

Further analysis indicated that the *lukED* gene was more frequently possessed by isolates from *agr* II group than by those from *agr* I group (94.6% versus 76.8%, *P* = 0.039), or mainly existed in ST188, ST7, ST764 and ST5 isolates, but not found in ST398 isolates (Table 2). Moreover, ST630 harbored the most frequency of *lukAB* (100.0%) compared to other STs, and no *pvl* was detected in ST7, ST764, ST5 and ST630 (Table 2).

**Table 2:** Comparison of toxin genes among major *agr* types or ST types

Toxin genes	No. of isolates positive for the toxin gene (%)									
	*Agr* type			ST type						
	*agr* I (*n* = 112)	*agr* II (*n* = 38)	*agr* III (*n* = 16)	ST59 (*n* = 9)	ST188 (*n* = 8)	ST398 (*n* = 8)	ST7 (*n* = 6)	ST764 (*n* = 6)	ST5 (*n* = 5)	ST630 (*n* = 5)
*lukED*	86 (76.8)	35 (94.6)	15 (93.8)	6 (66.7)	8 (100.0)	0	6 (100.0)	6 (100.0)	5 (100.0)	3 (60.0)
*lukM*	0	0	0	0	0	0	0	0	0	0
*hlgCB*	108 (96.4)	37 (97.4)	14 (87.5)	8 (88.9)	8 (100.0)	8 (100.0)	5 (83.3)	6 (100)	5 (100.0)	5 (100.0)
*lukAB*	81 (72.3)	29 (76.3)	8 (50.0)	5 (55.6)	6 (75.0)	2 (25.0)	4 (66.7)	5 (83.3)	4 (80.0)	5 (100.0)
*pvl*	12 (10.7)	0	1 (6.3)	3 (30.3)	1 (12.5)	1 (12.5)	0	0	0	0
*tst*	4 (3.6)	5 (13.2)	1 (6.3)	0	1 (12.5)	0	0	1 (16.7)	4 (80.0)	1 (20.0)
*sea*	21 (18.8)	4 (10.5)	5 (31.3)	3 (30.3)	2 (25.0)	1 (12.5)	0	0	1 (20.0)	0
*seb*	30 (26.8)	17 (44.7)	1(6.3)	7 (77.8)	4 (50.0)	1 (12.5)	0	4 (66.7)	1 (20.0)	0
*sec*	8 (7.1)	9 (23.7)	5 (31.3)	0	0	0	0	0	5 (100.0)	0
*sed*	7 (6.3)	6 (15.8)	0	1 (11.1)	0	0	0	1 (16.7)	0	0
*sej*	12 (10.7)	6 (15.8)	1 (6.3)	0	2 (25.0)	2 (25.0)	0	1 (16.7)	0	0
*see*	0	0	0	0	0	0	0	0	0	0
*seh*	9 (8.0)	0	9 (56.3)	0	1 (12.5)	0	0	0	1 (20.0)	0
*seg*	28 (25.0)	27 (71.1)	5 (31.3)	0	2 (25.0)	1 (12.5)	2 (33.3)	6 (100)	3 (60.0)	0
*sei*	19 (17.0)	25 (65.8)	3 (18.8)	0	0	0	1 (16.7)	4 (66.7)	3 (60.0)	0
*sem*	22 (19.6)	24 (63.2)	0	0	1 (12.5)	1 (12.5)	1 (16.7)	5 (83.3)	4 (80.0)	0
*sen*	18 (16.1)	18 (47.4)	1 (6.3)	0	1	0	1(16.7)	4(66.7)	4(80.0)	0
*seo*	16 (14.3)	17 (44.7)	1 (6.3)	0	0	0	1(16.7)	4(66.7)	4(80.0)	0

### Prevalence of PTSAg genes

The distribution of the PTSAg genes was shown in Table 1 and Fig. 1. The *seg* exhibited a relatively high prevalence (36.7%, 65/177), followed by *sei* (29.4%, 52/177), *sem* (28.8%, 51/177), *seb* (28.2%, 50/177), *sen* (22.6%, 40/177) and *seo* (21.5%, 38/177). The positive rates of the other PTSAg genes (*sea*, *sec*, *sed*, *seh*, *sej* and *tst*) were ranged from 7.3 to 18.6%, and no *see* was found. Statistical analysis showed *sen* (31.8%, 28/88 versus 13.5%, 12/89, *P* = 0.001) and *seo* (28.4%, 25/88 versus 14.6%, 13/89, *P* = 0.025) were more common in blood isolates than in wound isolates.

129 (72.9%, 129/177) isolates could be detected for possessing at least one of PTSAg genes, of which 41 (31.8%, 41/129, 28 from blood and 13 from wounds) harbored five or more PTSAg genes (high virulence gene content) (Fig. 1). The combination of the complete enterotoxin gene cluster *egc* (*seg*, *sei*, *sem*, *sen*, *seo*), coexisting with or without other PTSAg genes, was the most prevalent enterotoxin gene pattern, presenting in 22 blood isolates and 9 wound isolates (25.0%, 22/88 versus 10.1%, 9/89; *P* = 0.011). Moreover, the *sed-sej* combination was only presented in 4.0% (7/177) isolates.

Association between *agr* groups or major STs and PTSAg genes distribution was displayed in Table 2. The *sec* was markedly more common in *agr* II (*P* = 0.003) and *agr* III isolates (*P* = 0.014) than in *agr* I isolates. The *seh* was detected more often in isolates with *agr* III than in those with *agr* I and *agr* II (*P* < 0.001 each). Compared to *agr* I (*P* ≤ 0.039) and *agr* III isolates (*P* ≤ 0.007), the *agr* II isolates contain more the *seb*, *seg*, *sei*, *sem*, *sen* and *seo*. The most prevalent *egc* cluster was more commonly found in isolates belonging to *agr* II than those with *agr* I (34.2%, 13/38 versus 13.4%, 15/112, *P* = 0.004), or present commonly in ST5 (60.0%) and ST764 (50.0%) isolates, but was completely absent in ST59 and ST630 isolates. Additionally, we observed that *agr* II isolates contained higher number of enterotoxin genes (mean, 4.2) than *agr* I (mean, 1.7) and *agr* III isolates (mean, 2.0). ST5 and ST764 isolates carried higher frequency of PTSAg genes (mean, 6 and 5, respectively) compared to other STs. The *seb* was mainly present in ST59 strains (77.8%), followed by ST764 (66.7%) and ST188 (50.0%). In addition, the *sec* (100.0%) and *tst* (80.0%) were mainly found in ST5 strains. No enterotoxin gene was detected in ST630 isolates.

### Expression level of *lukED* among isolates with various genetic background

In the present study, our data confirmed the *S. aureus* isolates produced the highest expression amount of *lukED* at the late exponential growth phase (data not shown). Therefore, we chose this stage to study the expression of *lukED* in clinical isolates. Of the 63 *lukED*-positive *S. aureus* isolates typed by MLST, 46 selected randomly were grown to the late exponential growth phase for analyzing the mRNA expression of *lukED*. As shown in Fig. 1**,** an up to 260-fold difference was observed between the highest and the lowest *lukED* expressions strains, with the exception of two isolates expressing extremely low level of *lukED* [26× and 28×, both were PFGE39-*agr*I-CC20 (ST20/ST1281) blood isolates]. Compared to *lukED* expression level of strain Newman, 54.3% (25/46) isolates was 2 folds higher, whereas 41.3% (19/46) isolates was 2 folds lower (Fig. 1). Of note, two strains from wounds with the highest transcriptional levels of *lukED* all belonged to PFGE16-*agr*I-ST7, which were 23.0 (isolate 123) and 11.5 (isolate 752b) folds higher than that of Newman strain, respectively (Fig. 1). However, no significant differences were observed in the overall expression levels of *lukED* among the major ST types (ST5, ST7, ST188, ST630 and ST764, *P* = 0.072) or *agr* groups (*agr* I, *agr* II and *agr* III, *P* = 0.718) (Fig. 1).

## Discussion

Blood and wound *S. aureus* infections are common clinical diseases. Therefore, we investigated some major toxin (such as leukotoxin and PTSAg) genes existence for getting insight into the potential pathogenic ability of *S. aureus* from the two kinds of samples.

Although previous studies reported that each member of the leukotoxins has its distinct role in the pathogenesis of *S. aureus* by both in vitro and in vivo investigation [10, 31–35], to the best of our knowledge, this is the first study on the overall prevalence of this toxin family among clinical *S. aureus* isolates in China. Our data showed the prevalent rates of *hlgBC* (94.9%) and *lukED* (81.4%) were similar to those of previous reports [33, 36–41]. The *lukAB*, whose distribution is unknown due to lack of investigation in a large number of clinical strains, was carried by 67.8% of our isolates. There is a varying carriage of *pvl* among MRSA, ranging from 2.3 to 50.7% in China [42–45]. In the present study, a relatively low prevalence of *pvl*-positive isolates (7.9%) was found, which was in agreement with our previous data (6.6%) [46].

Regarding the PTSAg genes, Dramann et al. [5] has reviewed that approximately 80% of clinical *S. aureus* isolates carry an average of 5 to 6 genes, and the gene profiles varied remarkably among *S. aureus* strains. In this study, the overall positive rate of PTSAg genes was 72.9%, and a total of 59 PTSAg gene combinations were observed. However, due to only 13 PTSAg genes detected here, a much lower average carriage (mean, 3.3, 420/129) was found in the PTSAg gene-positive strains. A study from China showed *sea* was the most prevalent enterotoxin gene (41.53%) in *S. aureus* isolates from bacteraemia [47]. However, the positive rate of *sea* was only 15.9% in our blood strains, and had no significant difference between the strains from blood and wounds (15.9% versus 21.3%) (Table 1). This discrepancy is most likely caused by the difference in genetic backgrounds of strains [47, 48]. Although enterotoxin gene cluster *egc* has no connection with life-threatening infections, the possession of this operon may be conducive to the colonization of *S. aureus* and function in certain infections [49–51]. The total prevalence rate of 17.5% for the intact *egc* in this study (Fig. 1) resembled the results observed by Xie et al. [52] and Chao et al. [53]. However, we found a significant difference (24.7% versus 10.2%, *P* = 0.011) of this gene cluster carriage in our isolates from blood and wounds (Fig. 1), which indicated this cluster might link to the origins of isolates. Usually, *sed*-*sej* is located on plasmid pIB485, and the coexistence of both genes has been reported in some studies [53, 54]. Here, the fixed combination was only detected in 4% isolates, and 10.7% (19/177) isolates possessed *sej* or *sed* (Fig. 1). This uncharacterized combination of toxin genes indicated the diversity of yet-undescribed variants of mobile genetic element (MGE).

Previous studies presented that CC clones of *S. aureus* often display different toxin gene patterns [48, 53, 55]. For example, the toxin locus of *lukED* was present in CC1, CC5 and CC7 etc., but completely absent from CC22, CC30 and CC398 etc. [53, 55]. In this study, the distribution of *lukED* in CC isolates was basically in line with the previous reports, except 1 isolate with CC22 and 1 isolate with CC30 (Fig. 1). Previous data indicated that the *lukED* is located on a mobile pathogenicity island, vSaβ [5]. Therefore, we speculated the isolates with CC22 and CC30 obtained the *lukED* through the horizontal transfer of the vSaβ. Apart from *lukED* negative, ST398 isolates also harbor fewer PTSAg genes [56, 57]. This phenomenon was confirmed by our data in Fig. 1. Previous data indicated that the *egc* cluster was a common feature of CC5 isolates [58, 59]. The same phenomenon was found in our isolates **(**Fig. 1**)**. γ-hemolysin, a core genome-encoded leukotoxin, is highly conserved [3], and therefore can be detected in nearly all our *S. aureus* strains. Although *lukAB* is also located in the core genome, its locus is often disrupted by the insertion of a prophage [3]. This may explain the relatively lower prevalence of *lukAB* among each *S. aureus* lineage in this study, compared to that of γ-hemolysin-encoding gene *hlgCB* (Table 2, Fig. 1). Particular association had been observed between LukED-producing strains and *agr* II, as well as for TSST-1 and *agr* III isolates [21, 36]. However, our data only showed the correlation between *lukED* and *agr* II isolates. Besides, Fig. 1 displayed the PTSAg genes were preferable more common in *agr* II isolates. These carriage differences of toxin genes among isolates with different genetic backgrounds might be related to the heterogeneous nature of the infections and patients. In this study, the total number of isolates is not particularly large, which leaded to a relative small proportion (26.6%, 47/177) of MRSA strains and a few MRSA clones (Table 1 and Fig. 1). The asymmetrical distribution may influence the objective distribution of virulence determinants. This is one of the limitations of the present study.

The expression differences of immune evasion genes among strains may have vital influence for the pathogenesis of bacteria [55]. Previous studies exhibited that LukED plays an essential role in *S. aureus* infections [3, 10]. Therefore, we detected the transcription level of this leukotoxin in clinical isolates. The results of qRT-PCR revealed a marked strain-to-strain variation in *lukED* mRNA transcription, even in isolates with the same genetic characteristics. Consistent with the study on the production of PVL [12], we observed that *agr* types did not affect the *lukED* expressions significantly (Fig. 1). In addition, no remarkable associations were observed between major STs and the expression of this gene. Because only a limited number of ST or *agr* type isolates were included in this study, the different expressions of *lukED* attributed to various ST types or *agr* groups can’t be ruled out. And more comprehensive investigations of abundant isolates are needed to explore the association between *lukED* expression and different genetic background. In order to better verify the toxin’s role in bacterial pathogenesis, it is very important to study the relationship between the toxin expression level and the disease severity. In this study, we conducted a retrospective investigation designed only to understand the expression of *lukED* in *S. aureus* isolates from blood and wounds. If the correlation of the expression levels of *lukED* with the severity of infectious diseases is evaluated, it will provide a more convincing evidence to elucidate the function of LukED in the pathogenesis of *S. aureus*. This will be the research interest of the future study.

## Conclusions

In summary, this work exhibits the prevalence of leukotoxin and partial PTSAg genes in clinical *S. aureus* isolates from blood and wound in eastern region hospitals of China. Genotypic analysis illustrates a high genetic diversity of these isolates, and certain toxin genes, such as *lukED* and the *egc* cluster, may be lineage specific. In particular, *S. aureus* isolates belonging to ST764 and ST5, as well as *agr* II, were likely to harbor more PTSAg genes, which may suggest an alarming situation of infected patients. The different expression of *lukED* was found in clinical isolates, however, the association of this difference with the genetic backgrounds of isolates needs to be further unraveled.

## Additional file

Additional file 1: Sequences of primers used for PCR in this study. (DOCX 41 kb)

## Abbreviations

*agr*: Accessory gene regulator
CC: Clonal complex
CCs: Clonal complexes
HlgAB and HlgBC: Gamma (γ)-hemolysin
LPS: Lipopolysaccharide
LukAB/GH: Leukotoxin AB/GH
LukED: Leukotoxin ED
LukMFʹ: Leucocidin MFʹ
LukPQ: Leucocidin PQ
MGE: Mobile genetic element
MLST: Multilocus sequence typing
MRSA: Methicillin-resistant *Staphylococcus aureus*
PCR: Polymerase chain reaction
PFGE: Pulsed-field gel electrophoresis
PTSAgs: Pyrogenic toxin superantigens
PVL: Panton-Valentine leucocidin
qRT-PCR: Quantitative real-time PCR
*S. aureus*: *Staphylococcus aureus*
SCC*mec*: Staphylococcal cassette chromosome *mec*
SEs: Staphylococcal enterotoxins
STs: Sequence types
TSST-1: Toxic shock syndrome toxin-1
