# Supplementary material for: Leukotoxin and pyrogenic toxin Superantigen gene backgrounds in bloodstream and wound Staphylococcus aureus isolates from eastern region of China
Source: BMC Infect Dis. 2018 Aug 13;18:395. doi: 10.1186/s12879-018-3297-0 (PMC6090790; doi:10.1186/s12879-018-3297-0)
Supplement: Supplementary file 1 — Sequences of primers used for PCR in this study. (DOCX 41 kb) [file 12879_2018_3297_MOESM1_ESM.docx]

**Additional file**

**Leukotoxin and Pyrogenic Toxin Superantigen Gene Backgrounds in Bloodstream and Wound *Staphylococcus aureus* Isolates from Eastern Regions of China**

***Chunyan He ^1,#^, Su Xu ^2, #^, Huanqiang Zhao ^1^, Fupin Hu ^2^, Xiaogang Xu ^2^*, *Shu Jin ^3^, Han yang^1^, Fang Gong ^4^, Qingzhong Liu ^1,^****

*** Correspondence: *Qingzhong Liu***: [jiaodamedicine@foxmail.com](mailto:jiaodamedicine@foxmail.com)

**Additional file 1** Sequences of primers used for PCR in this study

| Primer | Sequence (5′-3′) | | | | Amplicon size (bp) | Reference |
| --- | --- | --- | --- | --- | --- | --- |
| Methicillin resistance gene | | | | | | |
| *mecA* | F-AAAATCGATGGTAAAGGTTGGC | | | | 533 | [18] |
|  | R-AGTTCTGCAGTACCGGATTTGC | | | |  |  |
| *mecC* | F-GCTCCTAATGCTAATGCA | | | | 304 | [19] |
|  | R-TAAGCAATAATGACTACC | | | |  |  |
| Leukotoxins gene | | | | | | |
| *lukED* | F-TGAAAAAGGTTCAAAGTTGATACGAG | | | | 269 | [21] |
|  | R-TGTATTCGATAGCAAAAGCAGTGCA | | | |  |  |
| *lukED*-RT | F-TCAATTGGCTGGGGTGTTGAGG | | | | 178 | this study |
|  | R-TACCACGCGCCAATAAAGGGA | | | | (Newman) |  |
| *hlgCB* | F-GCCAATCCGTTATTAGAAAATGC | | | | 938 | [22] |
|  | R-CCATAGAYGTAGCAACGGAT | | | |  |  |
| *lukAB* | F-TCACTTCTCCACCATACTTC | | | | 636 | this study |
|  | R-TATCAGCAGCAACGACTC | | | | (Newman) |  |
| *pvl* | F-ATCATTAGGTAAAATGTCTGGACATGATCCA  R-GCATCAACTGTATTGGATAGCAAAAGC | | | | 433 | [21] |
| 16S RNA-RT | F-TGAGATGTTGGGTTAAGTCCCGCA  R-CGGTTTCGCTGCCCTTTGTATTGT | | | | 188 | [30] |
|  |  | | | |  |  |
| PTSAg gene | | | | | | |
| *tst* | F-GCTTGCGACAACTGCTACAG | | | | 559 | [23] |
|  | R-TGGATCCGTCATTCATTGTTAT | | | |  |  |
| *sea* | F-GAAAAAAGTCTGAATTGCAGGGAACA | | | | 560 | [21] |
|  | R-CAAATAAATCGTAATTAACCGAAGGTTC | | | |  |  |
| *seb* | F-ATTCTATTAAGGACACTAAGTTAGGGA | | | | 404 |  |
|  | R-ATCCCGTTTCATAAGGCGAGT | | | |  |  |
| *sec* | F-GTAAAGTTACAGGTGGCAAAACTTG | | | | 297 |  |
|  | R-CATATCATACCAAAAAGTATTGCCGT | | | |  |  |
| *sed* | F-GAATTAAGTAGTACCGCGCTAAATAATATG | | | | 492 |  |
|  | R-GCTGTATTTTTCCTCCGAGAGT | | | |  |  |
| *see* | F-CAAAGAAATGCTTTAAGCAATCTTAGGC | | | | 482 |  |
|  | R-CACCTTACCGCCAAAGCTG | | | |  |  |
| *seg* | F-AATTATGTGAATGCTCAACCCGATC | | | | 642 |  |
|  | R-AAACTTATATGGAACAAAAGGTACTAGTTC | | | |  |  |
| *seh* | F- CGAAAGCAGAAGATTTACACG | | | | 495 | [24] |
|  | R-GACCTTTACTTATTTCGCTGTC | | | |  |  |
| *sei* | F-CTCAAGGTGATATTGGTGTAGG | | | | 576 | [21] |
|  | R-AAAAAACTTACAGGCAGTCCATCTC | | | |  |  |
| *sej* | F-TAACCTCAGACATATATACTTCTTTAACG | | | | 300 |  |
|  | R-AGTATCATAAAGTTGATTGTTTTCATGCAG | | | |  |  |
| *sem* | F-CTATTAATCTTTGGGTTAATGGAGAAC | | | | 300 |  |
|  | R-TTCAGTTTCGACAGTTTTGTTGTCAT | | | |  |  |
| *sen* | F-ATGAGATTGTTCTACATAGCTGCAAT | | | | 680 |  |
|  | R-AACTCTGCTCCCACTGAAC | | | |  |  |
| *seo* | F-AGTTTGTGTAAGAAGTCAAGTGTAGA | | | | 180 |  |
|  | R-ATCTTTAAATTCAGCAGATATTCCATCTAAC | | | |  |  |
| SCC*mec* typing | | | | |  | [25] |
| SCC*mec* I | F-GCTTTAAAGAGTGTCGTTACAGG | | | | 613 |  |
|  | R-GTTCTCTCATAGTATGACGTCC | | | |  |  |
| SCC*mec* II | F-CGTTGAAGATGATGAAGCG | | | | 398 |  |
|  | R-CGAAATCAATGGTTAATGGACC | | | |  |  |
| SCC*mec* III | F-CCATATTGTGTACGATGCG | | | | 280 |  |
|  | R-CCTTAGTTGTCGTAACAGATCG | | | |  |  |
| SCC*mec* IVa | F-GCCTTATTCGAAGAAACCG | | | | 776 |  |
|  | R-CTACTCTTCTGAAAAGCGTCG | | | |  |  |
| SCC*mec* IVb | F-TCTGGAATTACTTCAGCTGC | | | | 493 |  |
|  | R-AAACAATATTGCTCTCCCTC | | | |  |  |
| SCC*mec* IVc | F-ACAATATTTGTATTATCGGAGAGC | | | | 200 |  |
|  | R-TTGGTATGAGGTATTGCTGG | | | |  |  |
| SCC*mec* IVd | F-CTCAAAATACGGACCCCAATACA | | | | 881 |  |
|  | R-TGCTCCAGTAATTGCTAAAG | | | |  |  |
| SCC*mec* V | F-GAACATTGTTACTTAAATGAGCG | | | | 325 |  |
|  | R-TGAAAGTTGTACCCTTGACACC | | | |  |  |
| *agr* typing | |  |  |  | | [26] |
| pan *agr* | ATGCACATGGTGCACATGC | | | |  |  |
| *agr*I | GTCACAAGTACTATAAGCTGCGAT | | | | 439 |  |
| *agr*II | TATTACTAATTGAAAAGTGGCCATAGC | | | | 572 |  |
| *agr*III | GTAATGTAATAGCTTGTATAATAATACCCAG | | | | 321 |  |
| *agr*IV | CGATAATGCCGTAATACCCG | | | | 657 |  |
| MLST typing | |  |  |  | | [28] |
| *arcC* | F-TTGATTCACCAGCGCGTATTGTC | | | | 456 |  |
|  | R-AGGTATCTGCTTCAATCAGCG | | | |  |  |
| *aroE* | F-ATCGGAAATCCTATTTCACATTC | | | | 456 |  |
|  | R-GGTGTTGTATTAATAACGATATC | | | |  |  |
| *glpF* | F-CTAGGAACTGCAATCTTAATCC | | | | 465 |  |
|  | R-TGGTAAAATCGCATGTCCAATTC | | | |  |  |
| *gmk* | F-ATCGTTTTATCGGGACCATC | | | | 417 |  |
|  | R-TCATTAACTACAACGTAATCGTA | | | |  |  |
| *pta* | F-GTTAAAATCGTATTACCTGAAGG | | | | 474 |  |
|  | R-GACCCTTTTGTTGAAAAGCTTAA | | | |  |  |
| *tpi.* | F-TCGTTCATTCTGAACGTCGTGAA | | | | 402 |  |
|  | R-TTTGCACCTTCTAACAATTGTAC | | | |  |  |
| *yqiL* | F-CAGCATACAGGACACCTATTGGC | | | | 516 |  |
|  | R-CGTTGAGGAATCGATACTGGAAC | | | |  |  |

PTSAg, pyrogenic toxin superantigen; RT, used for qRT-PCR
